# Questioning the Role of Carotid Artery Ultrasound in Assessing Fluid Responsiveness in Critical Illness: A Systematic Review and Meta-Analysis

**DOI:** 10.1155/2024/9102961

**Published:** 2024-04-27

**Authors:** Samuel C. D. Walker, Adam C. Lipszyc, Matthew Kilmurray, Helen Wilding, Hamed Akhlaghi

**Affiliations:** ^1^Department of Emergency Medicine, St Vincent's Hospital, Melbourne, Victoria, Australia; ^2^Department of Anaesthesia and Acute Pain Medicine, St Vincent's Hospital, Melbourne, Victoria, Australia; ^3^Library Service, St Vincent's Hospital, Melbourne, Victoria, Australia; ^4^Department of Medical Education, University of Melbourne, Melbourne, Victoria, Australia

## Abstract

**Background:**

A noninvasive and accurate method of identifying fluid responsiveness in hemodynamically unstable patients has long been sought by physicians. Carotid ultrasound (US) is one such modality previously canvassed for this purpose. The aim of this novel systematic review and meta-analysis is to investigate whether critically unwell patients who are requiring intravenous (IV) fluid resuscitation (fluid responders) can be identified accurately with carotid US.

**Methods:**

The protocol was registered with PROSPERO on the 30/11/2022 (ID number: CRD42022380284). Studies investigating carotid ultrasound accuracy in assessing fluid responsiveness in hemodynamically unstable patients were included. Studies were identified through searches of six databases, all run on 4 November 2022, Medline, Embase, Emcare, APA PsycInfo, CINAHL, and Cochrane Library. Risk of bias was assessed using the QUADAS-2 and the Grading of Recommendations, Assessment, Development, and Evaluations (GRADE) guidelines. Results were pooled, meta-analysis was conducted where amenable, and hierarchical summary receiver operating characteristic models were established to compare carotid ultrasound measures.

**Results:**

Seventeen studies were included (*n* = 842), with 1048 fluid challenges. 441 (42.1%) were fluid responsive. Four different carotid US measures were investigated, including change in carotid doppler peak velocity (∆CDPV), carotid blood flow (CBF), change in carotid artery velocity time integral (∆CAVTI), and carotid flow time (CFT). Pooled carotid US had a pooled sensitivity, specificity, and AUROC with 95% confidence intervals (CI) of 0.73 (0.66–0.78), 0.82 (0.72–0.90), and 0.81 (0.78–0.85), respectively. ∆CDPV had sensitivity, specificity, and AUROC with 95% CI of 0.72 (0.64–0.80), 0.87 (0.73–0.94), and 0.82 (0.78–0.85), respectively. CBF had sensitivity, specificity, and AUROC with 95% CI of 0.70 (0.56–0.80), 0.80 (0.50–0.94), and 0.77 (0.78–0.85), respectively. Risk of bias and assessment was undertaken using the QUADAS-2 and GRADE tools. The QUADAS-2 found that studies generally had an unclear or high risk of bias but with low applicability concerns. The GRADE assessment showed that ∆CDPV and CBF had low accuracy for sensitivity and specificity.

**Conclusion:**

It appears that carotid US has a limited ability to predict fluid responsiveness in critically unwell patients. ∆CDPV demonstrates the greatest accuracy of all measures analyzed. Further high-quality studies using consistent study design would help confirm this.

## 1. Introduction

Intravenous fluid administration is the first-line therapy for patients presenting with acute circulatory failure [1]. While early fluid resuscitation reverses organ hypoperfusion and improves clinical outcomes, inappropriate fluids can increase morbidity and mortality [2–4]. A “fluid responder” is a patient who, upon receiving an intravenous fluid bolus, incurs an increase to their cardiac output. These patients are said to have “preload reserve,” where increasing their cardiac preload improves their stroke volume (SV) and ultimately cardiac output (CO). A “fluid nonresponder” is a patient whose stroke volume will not improve with further fluids, reflecting either an already optimised preload or advanced disease state. Reliable noninvasive and readily available tools to identify fluid responders in the setting of acute resuscitation remain clinically challenging as accurate and timely assessment of the fluid status requires either invasive or technically difficult procedures [5–7].

Ultrasound has had an increasingly important role in assessing fluid responsiveness in critically unwell patients in both emergency departments and intensive care units. There has been recent interest in the diagnostic accuracy of carotid artery ultrasound (US) as a noninvasive, accessible way to assess fluid responsiveness [8]. The seminal work conducted by Marik et al. [9] showed that carotid artery velocity time integral (CAVTI) had 94% sensitivity and 86% specificity in detecting fluid responsiveness in septic patients. Several studies have attempted to replicate the findings of this study in different clinical areas; however, heterogenous populations with small sample sizes make it difficult to draw meaningful conclusions.

Prior systematic review and meta-analyses of carotid ultrasound in determining fluid responsiveness have yielded promising results. Yao et al. [10] and Singla et al. [11] found that carotid US could be used to determine fluid responsiveness in surgical and ventilated patients. Similarly, Beier et al. [12] found that carotid US was a valid measure of fluid responsiveness in both healthy and unwell patients. Critically unwell patients have not been investigated in isolation. Patients in physiological extremis cannot be compared to elective and semielective surgical patients. These patients often require more intensive treatments, have longer stays in ICU, and have higher mortality rates than surgical patients [13, 14]. The aim of this novel systematic review and meta-analysis is to investigate whether critically unwell patients who are requiring intravenous (IV) fluid resuscitation (fluid responders) can be identified accurately with carotid US.

## 2. Methods

This review was performed following the Preferred Reporting Items for Systematic Reviews and Meta-Analysis of Diagnostic Test Accuracy (PRISMA-DTA) statement [15]. The protocol was registered with PROSPERO on the 30/11/2022 (ID number: CRD42022380284).

### 2.1. Study Selection and Inclusion Criteria

Studies were selected according to the PICOS statement.

#### 2.1.1. Patients and Setting

All studies which investigated the utility of carotid ultrasound as a measure of fluid responsiveness in critically unwell patients (shock of any kind and vasopressor requirement) were considered. Critically unwell was determined as patients who were requiring resuscitation as a result of some pathological process. Surgical studies were excluded unless they fulfilled this criterion independently of their surgery, e.g., appendicitis with septic shock. Studies were excluded if they had a portion of the cohort that was critically unwell, and the data were pooled and could not be separated. To avoid further confounding all noncritically ill subjects, healthy volunteers and children were also excluded.

#### 2.1.2. Index Tests and Reference Standards

Studies needed to have a reference standard for fluid responsiveness which was compared to a carotid ultrasound measure. Reference standards were any independent measure of measuring cardiac output or equivalent, e.g., cardiac index and stroke volume. Studies that investigated carotid US but had no reference standard were excluded.

#### 2.1.3. Comparison and Reference Standard

Reference standards were any independent measure of measuring cardiac output or equivalent, e.g., cardiac index and stroke volume. Studies that investigated carotid US but had no reference standard were excluded.

#### 2.1.4. Outcome and Target Condition

The target condition was fluid responsiveness. This was determined by measuring the reference standard before and after a fluid challenge. A fluid challenge could be achieved by providing the patient with a bolus of intravenous fluid or by providing them an “autotransfusion” by performing a passive leg raise (PLR). Patients were deemed fluid responsive if their cardiac output increased by a predetermined threshold. Studies which did not include an assessment of fluid responsiveness were excluded.

The primary outcome was the predictive value of carotid ultrasound measures to determine fluid responsiveness, expressed as an area under the receiver operating characteristics curve (AUROC). Studies which did not include AUROC, sensitivity, and specificity or studies in which these values could not be calculated were excluded.

#### 2.1.5. Study Design and Report Characteristics

Only prospective studies were included. Animal studies were excluded, as were the following publication types: books, chapters, conference abstracts, comments, dissertations, editorials, guidelines, letters, news, notes, policy statements, and study protocols. Papers in languages other than English were excluded.

### 2.2. Information Sources

Publications were identified through searches of the following six bibliographic databases, all run on 1 December 2023: Ovid Medline(R) ALL 1946–December 01, 2023; Embase 1974–2023 December 01 (Ovid); Ovid Emcare 1995–2022 week 43; APA PsycInfo 1806–December week 1, 2023 (Ovid); CINAHL (EBSCOhost); and Cochrane Library (Wiley). Two trial registries were searched on 1 December 2023, namely, Clinicaltrials.gov and Australia New Zealand Clinical Trials Registry (ANZCTR). Reference lists of included studies were examined for additional publications.

### 2.3. Search Strategy

Search strategies were developed by a medical librarian (HW) in consultation with a topic expert (SW), who provided a “gold set” of 10 relevant publications identified during scoping searches. These were checked for search terms and used to validate search strategies. Further search terms were identified through text mining in PubMed PubReminer [16] using the query “ultrasonography AND carotid AND fluid.” Search terms retrieved through text mining were extensively tested for usefulness and relevance in Ovid Medline to develop the final search strategy.

**Figure 1:**
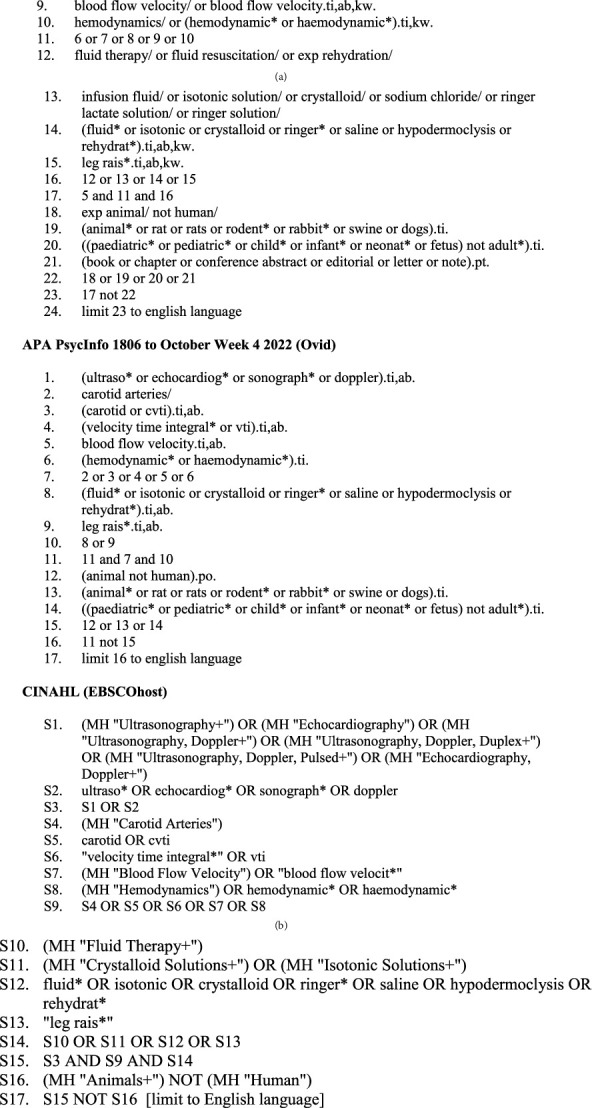
Search strategies.

Final search strategies combined the general concepts of ultrasonography AND carotid velocity time integral AND fluid responsiveness using a combination of subject headings and text words. An initial search was developed for Ovid Medline and then adapted for other databases adjusting subject headings and syntax as appropriate (Figure 1). Search syntax used in the Ovid databases was adapted for CINAHL (EBSCOhost) and Cochrane (Wiley) using the Polyglot Search Translator [17]. Trial registries were searched using the strategy “ultrasound AND carotid AND fluid.”

### 2.4. Study Selection

Database search results were exported to EndNote bibliographic management software (Clarivate Ltd, U.S.) and duplicates removed. In accordance with eligibility criteria records, these were screened on the publication type by HW within EndNote and book sections, comments, dissertations, and letters were excluded. All remaining records were loaded into Covidence systematic review software (Veritas Health Innovation Ltd) for screening on title and abstract. Records were independently screened on title and abstract in Covidence by two reviewers, SW and AL, and conflicts were resolved by HA. Full text records were retrieved for the remaining records.

### 2.5. Data Collection, Management, and Definitions

Data from all relevant studies were collected in the following domains: (1) study characteristics including author, year of publish, mean age, setting, sampling, percentage of fluid responders, percentage mechanically ventilated, type of fluid challenge, reference standard and threshold, carotid measure, and equipment used; (2) diagnostic performance, including sensitivity, specificity, true positives (TPs), true negatives (TNs), false positives (FPs) and false negatives (FNs), and AUROC and 95% confidence interval (CI). Where studies performed more than one carotid measure or more than one cohort of fluid challenges, these results were independently used for their relevant analysis. A true positive was defined as a significant change in carotid US measure in response to a fluid challenge as well a positive change in cardiac output or equivalent as per the predetermined reference standard. A true negative was deemed a nonresponder by the reference standard and a nonsignificant carotid US measure. A false positive was considered diagnosis of fluid responsive for the carotid US measure that was not confirmed by the reference standard. A false negative was considered not a fluid responder by carotid US which was diagnosed by the reference standard.

### 2.6. Assessment of Bias and Evaluation of Evidence Quality

The quality of the studies included in the review was assessed using the QUADAS-2 [18]; this was independently undertaken by two authors (SW and AL) with disagreements (12%) settled by consensus. The overall certainty was assessed using the Grading of Recommendations, Assessment, Development, and Evaluations guidelines [19, 20]. Overall certainty in the pooled sensitivity and specificity were categorised as high, moderate, low, or very low using the GRADEpro guideline development tool [21].

### 2.7. Statistical Analysis

The statistical analysis was undertaken using STATA 17.0 (StataCorp LLP, U.S.). Pooled sensitivity and specificity were calculated for each carotid measure. In instances where the TP, TN, FP, or FN values were not published or available in supplemental data, these were calculated using a 2-way contingency table analysis [22]. Meta-analysis was conducted in line with current standards [23] and side-by-side forest plots were used to examine variability between studies. The hierarchical summary receiver operator characteristic curve (HSROC) was plotted for carotid US measures in cases where five or more cohorts were available for analysis. The following values were pooled using a bivariate random effects model: sensitivity, specificity, positive likelihood ratio, negative likelihood ratio, and diagnostic odds ratio (DOR). Heterogeneity was also examined using the *I*^2^ statistic (whereby ≧75% suggested significant statistical heterogeneity between studies) in complement with inspection of forest plots and the HSROC models where applicable. The contribution of threshold effect was evaluated by Spearman's coefficient (for which a value ≥ 0.6 suggested a threshold effect) and review of the HSROC model shape. Deek's funnel plot asymmetry test was used to assess for publication bias. A metaregression was utilized to assess subgroup bias; this could only be performed for pooled carotid US due to insufficient numbers within the subgroups. Metaregression was used to assess the effects of the following dichotomous variables: index test threshold (10% vs 15%), reference measurement (“gold standard”-LVOT VTI/PAC thermodilution vs. “non-gold standard”-pulse contour cardiac output (PiCCO), FloTrac™, noninvasive cardiac output monitor (NICOM)), type of fluid challenge (IV fluid vs. PLR), and severity of sepsis (septic shock vs. sepsis).

## 3. Results

### 3.1. Study Selection and Study Characteristics

**Figure 2:**
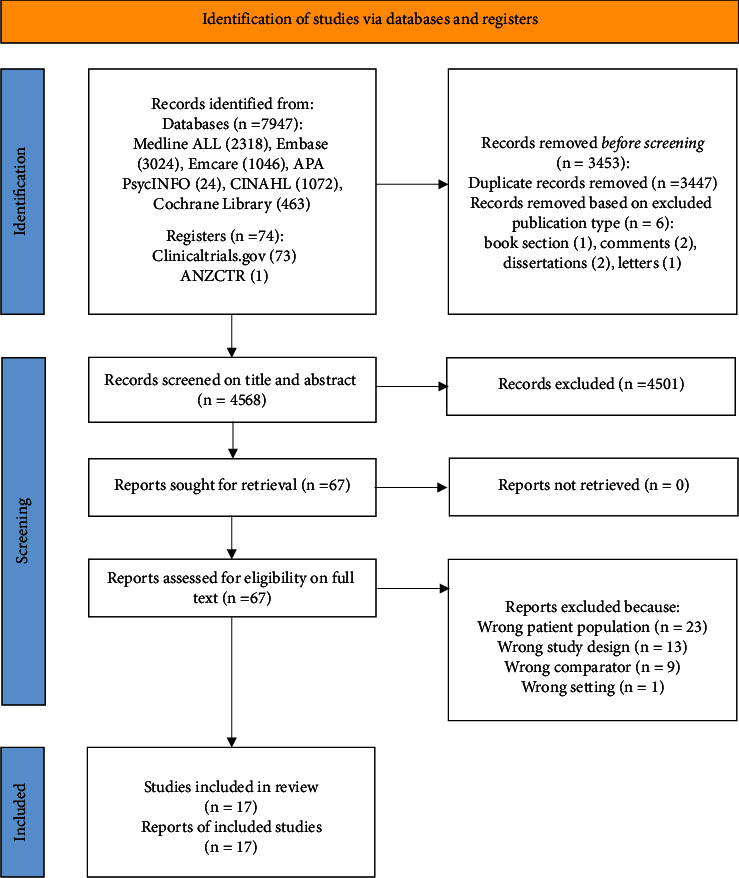
PRISMA 2020 flow diagram (as at 1 December 2023) [24].

The study selection methodology is summarised in Figure 2. 7947 records were identified from database and register searches, 3453 duplicates were removed, and 6 records excluded based on publication type. 4568 records were screened on title and abstract and 4501 excluded as irrelevant. 67 full-text reports were retrieved, assessed for eligibility, and 51 reports were excluded. 17 studies were included in the review and meta-analysis. In total, 860 patients underwent 1092 fluid challenges, of which 460 (42.1%) were fluid responsive.

Table 1 shows the characteristics of the 17 included studies. The majority of studies were conducted in ICU, with one study conducted in an emergency department [34]. There were multiple reasons for hemodynamic instability. The majority were unspecified/heterogeneous [9, 25, 29, 32, 34, 35, 37, 39] or septic shock [26, 30, 31, 33, 36, 40], with a minority of studies having cohorts of patients with haemorrhagic shock [27] or cardiogenic shock [38]. A fluid challenge was administered either by crystalloid bolus [27–31, 34, 37, 40], passive leg raise (PLR) [25, 26, 32, 38, 39], or a combination of the two [9, 33, 35, 36]. Crystalloid volume was determined by weight (6-7 ml/kg) or a predetermined value (200 ml–500 ml). The reference standards most commonly used were left ventricular outflow tract (LVOT) velocity time integral (VTI) [26, 27, 31, 33, 34, 37, 38] and noninvasive cardiac output monitor (NICOM) (Cheetah Medical, Inc) [9, 25, 28, 29], with some studies used pulmonary artery catheter (PAC) [32, 36, 40], pulse contour cardiac output (PiCCO) (PULSION Medical Systems AG, Munich, Germany) [30, 35], and FloTrac (Edwards Lifesciences, Irvine, CA, USA) [39].

The threshold for fluid responsiveness measured against the reference standard was measured by a 10–15% increase of reference standard after a fluid challenge for all studies. The severity of illness of patient cohorts was poorly documented. Patient cohorts who were mechanically ventilated varied significantly, ranging between 0% and 100%. Four different carotid measures were used including change in carotid doppler peak velocity (∆CDPV) [27, 29–31, 36, 37, 40], carotid blood flow (CBF) [9, 28, 33, 34, 38], change in carotid artery velocity time integral (∆CAVTI) [9, 26, 33, 35, 37], and carotid flow time (CFT) [25, 28, 32, 39, 40]. One study used carotid time-averaged mean velocity (TAMEAN) [35]. Two studies performed two subgroup analysis with two carotid measures [28, 37], and one study ran two cohorts one with PLR and one with IVF [33].  Table 2 details the US equipment used for included studies.

### 3.2. Risk of Bias and Quality of Evidence

**Figure 3:**
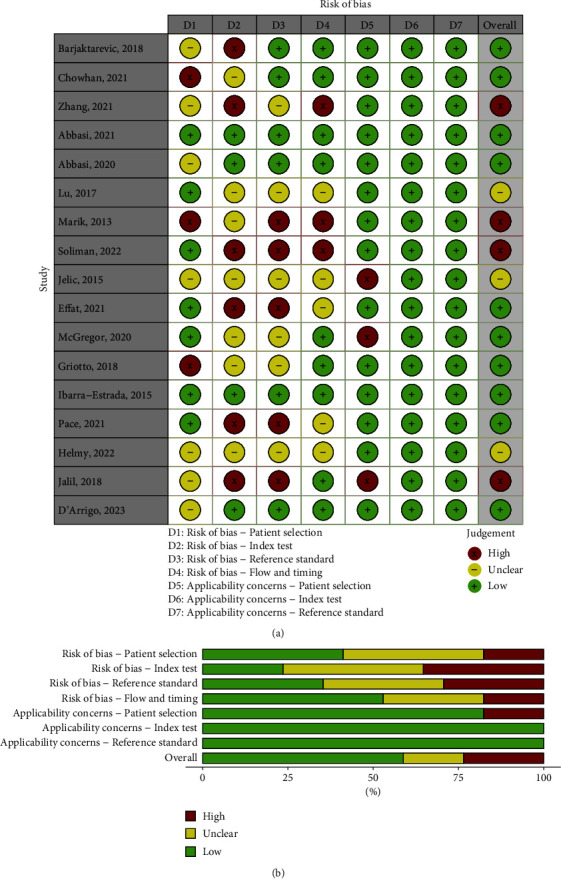
QUADAS-2 risk of bias and applicability concerns.

Quality assessment of included studies was performed using the QUADAS-2 (Figure 3). Generally, the risk of bias of studies was significant. Most studies performed convenience recruitment, citing the impracticalities of continuous or random recruitment in busy, unpredictable critical care environments. Most studies had similar exclusion criteria (unable to tolerate PLR and carotid stenosis); however, some studies excluded common comorbidities which may have led to a skewed cohort. For example, Chowan et al. [26] excluded all patients with a body mass index (BMI) > 30 or if patients had any valvular heart disease or “cardiac stenosis.” Another common issue was the lack of blinding between the index and the reference scans. No studies set a predetermined threshold for the index test which would be deemed as a “positive test;” these were all established post hoc. The quality of the reference standard was generally high (Figure 3). The GRADE evidence is provided in Table 3, and it found that for ∆CDPV and CBF had low accuracy for sensitivity and specificity.

### 3.3. Performance of Carotid Ultrasound in Predicting Fluid Responsiveness

**Figure 4:**
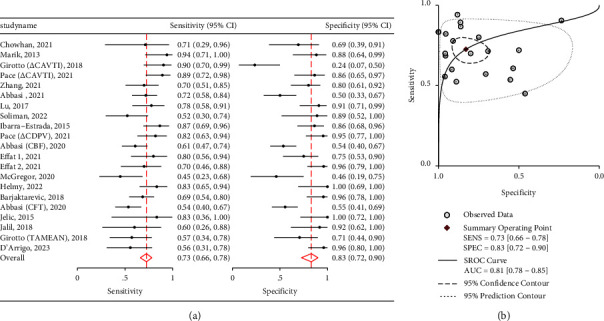
(a) Twin forest plots for pooled carotid US measures when assessing fluid responsiveness in hemodynamically unstable patients. (b) AUROC and HSROC model for pooled carotid US measures.

Seventeen studies were considered for the meta-analysis. The primary outcome was the efficacy of carotid ultrasound in predicting fluid responsiveness in critically unwell patients.  Figure 4(a) shows a twin forest plot, illustrating the pooled carotid ultrasound figures as follows: sensitivity and specificity of 0.73 (95% CI 0.66–0.78) and 0.83 (95% CI 0.72–0.90), respectively.  Figure 4(b) shows a pooled AUROC of 0.81 (95% CI 0.78–0.85) and a HSROC model for pooled US measures. It had a positive likelihood ratio of 4.24 (2.49 and 7.23) and a negative likelihood ratio of 0.33 (0.25 and 0.43).

**Figure 5:**
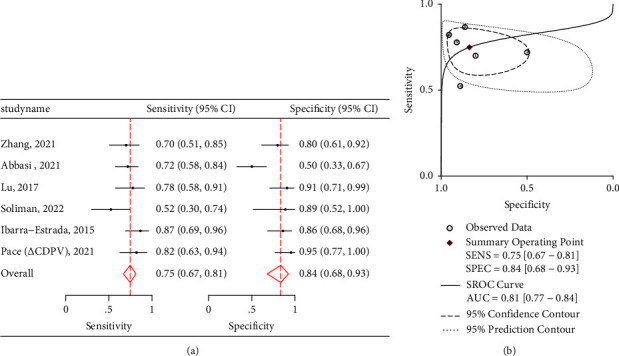
(a) Twin forest plots for ∆CDPV when assessing fluid responsiveness in hemodynamically unstable patients. (b) HSROC and AUROC graph for ∆CDPV.

**Figure 6:**
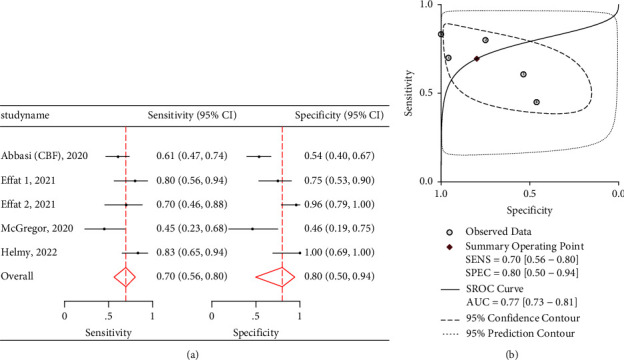
(a) Twin forest plots for CBF when assessing fluid responsiveness in hemodynamically unstable patients. (b) HSROC and AUROC graph for CBF.

Two carotid index parameters were amenable to ad-hoc meta-analysis (∆CDPV and CBF) which are shown in Figures 5 and 6. ∆CDPV had a pooled sensitivity of 0.72 (95% CI: 0.64–0.80) and specificity of 0.87 (95% CI: 0.73–0.94) (Figure 5(a)). ∆CDPV had a pooled AUROC of 0.82 (95% CI: 0.78–0.85) (Figure 5(b)). It had a positive likelihood ratio of 5.48 with wide confidence intervals (2.52–11.90) and a negative likelihood ratio of 0.31 (0.23 and 0.43) as shown in Table 4.

**Figure 7:**
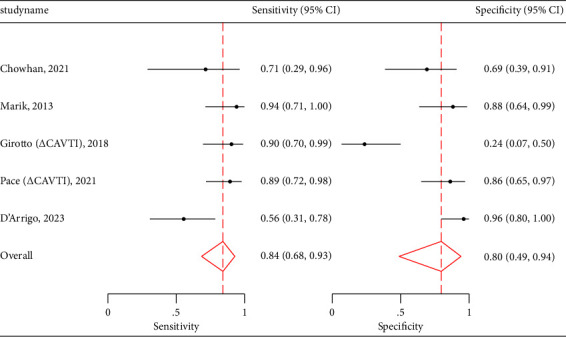
CAVTI paired forest plots.

**Figure 8:**
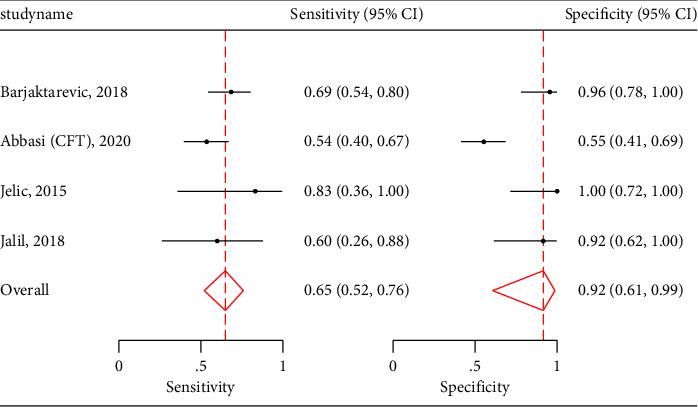
CFT paired forest plots.

CBF demonstrated a pooled sensitivity of 0.70 (95% CI: 0.56–0.80), specificity of 0.80 (95% CI: 0.50–0.94) (Figure 6(a)), and an AUROC of 0.77 (95% CI: 0.73–0.81) (Figure 6(b)). It had positive likelihood ratio of 2.00 (1.56 and 2.56) and negative likelihood ratio of 0.45 (0.34 and 0.60). Table 4 also shows data for the remaining carotid US measures, including pooled sensitivities, specificities, positive likelihood ratios, and negative likelihood ratios. Unfortunately, ∆CAVTI and CFT did not have enough studies to perform a regression analysis; however, their pooled sensitivities and specificities can be viewed in Table 5 and their paired forest plots in Figures 7 and 8. Given TAMEAN was only used in one study, no further analysis was performed. 

Table 6 details the subgroup metaregression analysis performed. Taking a significant *p* value to be ≤ 0.05, several variables found significance. Specifically, it was found that studies which used the reference gold standard measures (LVOT VTI and PAC) had significantly higher specificities in detecting fluid responsiveness than studies which used less widely validated measures of CO. Passive leg raise had significantly higher specificity than IV crystalloid, and studies which investigated sepsis/septic shock cohorts had a statistically significant higher sensitivity although this appears to be an insignificant number practically with sensitivities only differing by 0.01. Table 6 shows data for each carotid US measure, including pooled sensitivities, specificities, positive likelihood ratios, and negative likelihood ratios.

### 3.4. Heterogeneity

**Figure 9:**
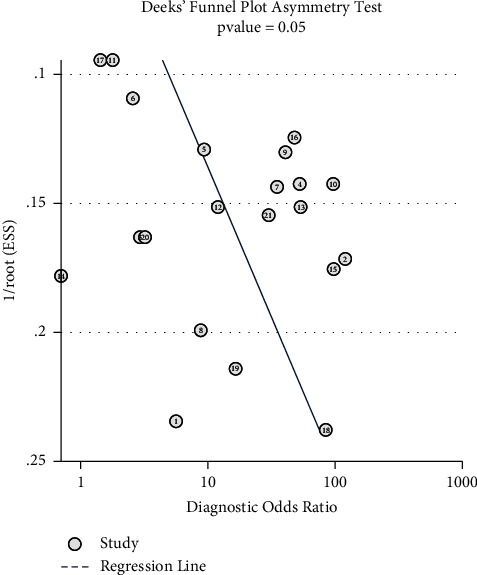
Deek's funnel plot asymmetry test.

*I* ^2^ values for pooled sensitivity and specificity were 48.6% and 68.0%, respectively. This indicates that there may be moderate to substantial heterogeneity between studies. Within the studies which examined, only ∆CAVTI and CBF heterogeneity was as follows: ∆CAVTI *I*^2^ values for sensitivity and specificity were 35.6% and 59.7%, respectively, and CBF *I*^2^ values for sensitivity and specificity were 55.2% and 72.4%, respectively; this represents high heterogeneity.  Figure 9 illustrates a statistically significant asymmetric Deek's funnel plot with a *p* value of 0.05, indicating high likelihood of publication bias.

## 4. Discussion

This novel systematic review and meta-analysis reviewed the literature aiming to assess the diagnostic accuracy of carotid US in predicting fluid responsiveness in critically unwell patients. Seventeen studies were included in the review. We conclude that carotid US measures shows a moderate sensitivity and a high specificity in predicting fluid responsiveness in critically unwell patients. However, these results should be interpreted with caution due to the high heterogeneity among the existing studies and the low confidence in the accuracy findings based on the GRADE assessment.

Previous reviews investigating carotid ultrasound in well patients and surgical patients have shown promising results, with pooled sensitivities of 0.83–0.85 and specificities of 0.86–0.89 with a AUROC of 0.894–0.927 for ∆CDPV in predicting fluid responsiveness [10, 11]. However, our review of critically unwell patient demonstrates that carotid US measures are less reliable in this population compared to these prior findings. Notably, the sensitivity of ∆CDPV in our review was significantly lower at 0.72, indicating a decreased ability to accurately identify fluid responders among critically ill patients. Whilst specificity of 0.87, the positive likelihood ratio of 5.48 and negative likelihood ratio of 0.31 for ∆CDPV were similar to previous reviews; the lower sensitivity represents a key difference in the diagnostic performance of carotid US in this specific patient population.

In comparison to other ultrasound measures, carotid US was midrange in its ability to diagnose fluid responsiveness in critically unwell patients. Carotid US was significantly inferior to LVOT VTI which has sensitivity and specificity of 0.88 and 0.95, respectively, in septic shock patients [41]. It was also outperformed by internal jugular vein US in acutely unwell patients which had pooled sensitivities and specificities of 0.82 and 0.78, respectively [42]. It performed similarly in sensitivity to IVC diameter, 0.71, which was deemed unreliable as a measure of fluid responsiveness. Carotid US did have, however, a more favourable sensitivity to IVC diameter (0.71) [43].

Authors have hypothesized as to why carotid artery may be suboptimal when compared to the left ventricular outflow tract in acutely unwell patients. There has been a suggestion that the carotid artery may play an important part of cerebral blood flow autoregulation [44], meaning that changes in cardiac output are not accurately identified at the level of the carotid artery. This effect may be further exacerbated in shocked and critically unwell patients reflecting the decreased diagnostic utility of our review when compared to other measures of fluid responsiveness.

Most patient cohorts within this review were based in ICU. This in unsurprising as it has the highest density of hemodynamically unstable patients with clinicians having more time with the patient allowing serial carotid US measurements. Interestingly, the only emergency department study (McGregor et al. [34]) demonstrated the lowest sensitivity and specificities among included studies, 0.45 and 0.46, respectively. This group of patients had received less intravenous fluid (compared to ICU patients); intuitively, this would suggest they would be more likely to be on the descending portion of the Frank–Starling curve and theoretically be more sensitive to fluid therapy when compared to ICU patients; however, this was not seen. Further studies are needed in emergency department settings to test carotid US diagnostic utility in this context.

A recent meta-analysis investigating factors affecting fluid responsiveness and how they are related to operative performance demonstrated that variables such as the volume of intravenous fluid, choice of hemodynamic variable, noradrenaline dosing, and duration of end expiratory hold can significantly impact operative performance [45]. These results have important clinical implications as failing to account for such factors could lead to inaccurate assessment of fluid responsiveness and inappropriate administration or withholding of fluid therapy. Our review compliments the findings of this review by illustrating that carotid US's ability to detect fluid responsiveness requires a nuanced application and caution in critically unwell patients.

One of the most significant variabilities between the studies was the threshold which deemed a carotid US measure to be “fluid responsive.” None of the studies set a predetermined value, rather the cutoff was decided post hoc. Fluid responders according to the index test ranged from a 7% to a 23% increase, making it very difficult for clinicians to determine where fluid responsiveness lies with carotid US. Another area of variability between studies was the choice of reference standard. Most studies used either LVOT VTI or PAC to identify fluid responders, widely accepted as accurate ways of assessing cardiac output. Girotto et al. [35] and Lu et al. [30] used PiCCO™ which is a device that utilizes transpulmonary thermodilution. Several studies have shown PiCCO™ to be reliable when compared to PAC [46, 47]. Jalil et al. [39] used FloTrac™ which is a noninvasive device and has shown variable results in its ability to accurately identify changes in cardiac output. Three studies [28, 29, 39] used NiCOM, a noninvasive monitor which estimates cardiac output. Some studies showed that it can be a reliable measure [48], whilst others have shown that NiCOM cannot be used to estimate cardiac output, notably in critically ill patients [49].

### 4.1. Limitations

This study had several limitations. One limitation was that only two carotid US measures were amenable to meta-analysis. Unfortunately, CFT, ∆CAVTI, and carotid TAMEAN did not have enough data to perform the meta-analysis. Another limitation was the heterogeneity between the studies. Our analysis showed that there was a moderate interstudy heterogeneity. The absence of a uniform cut-off for carotid US measures limits clinical applicability.

There is an opportunity for future research investigating the use of carotid ultrasound in hemodynamically unstable patients.

Prospective investigators should consider using ∆CDPV as their carotid ultrasound measure, in a homogeneous patient population (for example septic shock), with a predefined cutoff for their carotid US measure.

## 5. Conclusion

We conclude that the available data from existing literature carotid US is moderately effective at diagnosing fluid responsiveness in critically unwell patients. However, our results suggest that carotid US is less accurate acutely unwell patients compared to surgical cohorts. Our study showed moderate to high heterogeneity within the literature and low accuracy confidence when applying the GRADE framework. Clinicians should use carotid US in critically unwell patients with caution. Despite the limitations, this systematic review and meta-analysis offers the most rigorous and comprehensive evaluations of the existing literature.

## Figures and Tables

**Table 1:** Characteristics of the included studies.

Study, year	Setting	Sampling	Sample size (% fluid responders)	Patient group	MV	Fluid challenge content	Fluid challenge volume	Ref. St. measure	Ref. St. threshold (%)	Carotid measure
Barjaktarevic et al., 2018 [25]	ICU	Convenience	77 (70.1%)	Undifferentiated shock	59%	PLR	n/a	SV via NICOM™	10	CFT
Chowhan et al., 2021 [26]	ICU	Convenience	20 (septic shock group)	Control, sepsis, and septic shock	100%	PLR	n/a	SV via LVOT VTI	>15	∆CAVTI
Zhang et al., 2021 [27]	ICU	Convenience	60 (50%)	Traumatic haemorrhagic shock	100%	0.9% sodium chloride	250 ml	CO via LVOT VTI	≥15	∆CDPV
Abbasi et al., 2020 [28]	ICU	Convenience	112 (50%)	Acute circulatory failure within the first 72 hours	0%	0.9% sodium chloride	500 ml	NICOM™ CI	≥10	CBF
Abbasi et al., 2021 [29]	ICU	Convenience	86 (58%)	Acute circulatory failure within the first 72 hours	0%	0.9% sodium chloride	500 ml	NICOM™ CI	≥10	∆CDPV
Lu et al., 2017 [30]	ICU	Convenience	49 (55%)	Septic shock	NS	0.9% sodium chloride	200 ml	CO and CI index via PiCCO™	≥10	∆CDPV
Marik et al., 2013 [9]	ICU	Not stated	34 (53%)	Hemodynamic instability (64.7% with septic shock)	56%	PLR + 0.9% sodium chloride	500 ml	SV via NICOM™	≥10	∆CAVTI
Soliman et al., 2022 [31]	ICU	Not stated	30 (70%)	Septic shock	100%	Crystalloid	7 ml/kg	CO via LVOT VTI	≥15	∆CDPV
Jelic et al., 2015 [32]	ICU	Not stated	17 (29%)	Shock	NS	PLR	n/a	PAC	≥10	CFT
Effat et al., 2021 [33]	ICU	Not stated	44 (45%)	Sepsis ± shock	46%	PLR + 0.9% saline	6 ml/kg	LVOT VTI	≥15	CBF
McGregor et al., 2020 [34]	ED	Convenience	33 (61%)	Patients which required an IV fluid bolus	0%	Crystalloid	250–500 ml	SV via LVOT VTI	≥10	CBF
Girotto et al., 2018 [35]	ICU	Not stated	VTI 60 (67%)	PiCCO2 device in situ, decision to PLR not stated	94%	PLR + 0.9% sodium chloride	500 ml	Cardiac index via PiCCO™	≥10 on pulse contour	∆CAVTI
Ibarra-Estrada et al., 2015 [36]	ICU	Convenience	19 patients 59 fluid challenges (51%)	Septic shock	100%	PLR + crystalloid	7 ml/kg	PAC	≥15	∆CDPV
Pace et al., [37]	ICU	Convenience	50 (56%)	Hemodynamically unstable	100%	Crystalloid	7 ml/kg	SV via aortic VTI	≥15	∆CAVTI + ∆CDPV
Helmy et al., 2022 [38]	ICU	Not stated	40 (75%)	Cardiogenic shock	78%	PLR	n/a	CO via LVOT VTI	≥10	CBF
Jalil et al., 2018 [39]	ICU	Not stated	22 (45%)	Patients which require IV fluid bolus	82%	PLR	n/a	SV via FloTrac™	≥15	CFT
D'Arrigo et al., 2023 [40]	ICU	Consecutive	18 patients 44 fluid challenges (43.2%)	Septic shock	100%	Crystalloid	500 ml	Cardiac index via thermodilution	>15	∆CDPV + CFT

MV = mechanically ventilated, Ref. St. = reference standard, ICU = intensive care unit, ED = emergency department, PLR = passive leg raise, SV = stroke volume, NICOM = noninvasive cardiac output monitor, CFT = carotid flow time, ∆CAVTI = change in carotid artery velocity time integral, LVOT VTI = left ventricular outflow tract velocity time integral, ∆CDPV = change in carotid Doppler peak velocity, CBF = carotid blood flow, CO = cardiac output, PAC = pulmonary artery catheter, PiCCO = pulse contour cardiac output, CI = cardiac index, IV = intravenous.

**Table 2:** US equipment for the included studies.

Study	Equipment	Probe	Frequency (MHz)
Barjaktarevic et al. [25]	LOGIQ e, GE Healthcare	Linear	—
Chowhan et al. [26]	IMAGIC Agile, Kontron Medical	Phase	—
Zhang et al. [27]	Mindray M9 Diagnostic, US	Linear	8–12
Abbasi et al. [28]	Sonosite edge ultrasound	Linear	6–13
Abassi et al. [29]	Sonosite edge ultrasound	Linear	6–13
Lu et al. [30]	Sonosite	—	12
Marik et al. [9]	LOGIQ e; GE Healthcare	Linear	7–12
Soliman et al. [31]	GE LOGIQ™ P9- South Korea, FUJIFILM SonoSite M-Turbo®- Malaysia	Linear	5–10
Jelic et al. [32]	—	—	—
Effat et al. [33]	P4–2 siemens acuson ×300, siemens medical system	Linear	—
McGregor et al. [34]	Sonosite EDGE	—	—
Girotto et al. [35]	CX50 (Philips Healthcare)	Linear	5–12
Ibarra-Estrada et al. [36]	Sonosite micromaxx system	Linear	5–10
Pace et al. [37]	MyLab60	Linar	5–10
Helmy et al. [38]	Phillips HD11 XE	Phased	2.5
Jalil et al. [39]	FujuFilm sonosite	Linear	—

**Table 3:** (a) GRADE evidence profile for ∆CDPV. (b) GRADE evidence profile for CBF.

Outcome	№ of studies (no of patients)	Study design	Factors that may decrease certainty of evidence					Effect per 1,000 patients tested			Test accuracy CoE
			Risk of bias	Indirectness	Inconsistency	Imprecision	Publication bias	Pretest probability of 10%	Pretest probability of 20%	Pretest probability of 50%	
(a) Question: should ∆CDPV be used to diagnose fluid responsiveness in critically unwell patients?											
True positives (patients with fluid responsiveness)	7 studies 297 patients	Cross-sectional (cohort type accuracy study)	Serious^a^	Serious^b^	Not serious	Not serious	None	72 (63–80)	144 (126–160)	360 (315–400)	⊕⊕○○ low
False negatives (patients incorrectly classified as not having fluid responsiveness)								28 (20–37)	56 (40–74)	140 (100–185)	

True negatives (patients without fluid responsiveness)	7 studies 297 patients	Cross-sectional (cohort type accuracy study)	Serious^a^	Serious^c^	Not serious	Not serious	None	783 (657–846)	696 (584–752)	435 (365–470)	⊕⊕○○ low
False positives (patients incorrectly classified as having fluid responsiveness)								117 (54–243)	104 (48–216)	65 (30–135)	

Sensitivity	0.72 (95% CI: 0.63–0.80)										

Specificity	0.87 (95% CI: 0.73–0.94)										

Prevalences	10%				20%				50%		

(b) Question: should CBF be used to diagnose fluid responsiveness in critically unwell patients?											
True positives (patients with fluid responsiveness)	5 studies 173 patients	Cross- sectional (cohort type accuracy study)	Serious	Serious	Not serious	Not serious	None	70 (56–80)	140 (112–160)	350 (280–400)	⊕⊕○○ low
False negatives (patients incorrectly classified as not having fluid responsiveness)								30 (20–44)	60 (40–88)	150 (100–220)	

True negatives (patients without fluid responsiveness)	5 studies 173 patients	Cross- sectional (cohort type accuracy study)	Serious	Serious	Not serious	Not serious	None	720 (450–846)	640 (400–752)	400 (250–470)	⊕⊕○○ low
False positives (patients incorrectly classified as having fluid responsiveness)								180 (54–450)	160 (48–400)	100 (30–250)	

Sensitivity	0.70 (95% CI: 0.56–0.80)										

Specificity	0.80 (95% CI: 0.50–0.94)										

Prevalences	10%				20%				50%		

Explanations: ^a^several studies failed to identify the independence of the index test and reference standard, i.e., blinding. ^b^The recruitment methodology was not specified in several studies. Some studies failed to exclude patients with conditions (aortic stenosis) where carotid US may be unreliable. ^c^Some studies excluded patients with heart failure and other comorbidities, which are common in critically unwell. This may impact its generalisability.

**Table 4:** Diagnostic accuracy of carotid ultrasound measures to predict fluid responsiveness.

Analysis	Source	Positive/total analysed	Sensitivity (95% CI)	Specificity (95% CI)	Positive likelihood ratio (95% CI)	Negative likelihood ratio (95% CI)
∆CDPV	Zhang et al. [27], Abbasi et al. [29], Lu et al. [30], Soliman et al. [31], Ibarra-Estrada et al. [36] Abbasi et al. [29], Pace et al. [37], D'Arrigo et al. [40]	148/297	0.72 (0.63, 0.80)	0.87 (0.73, 0.94)	5.48 (2.52, 11.9)	0.31 (0.23, 0.43)
CBF	Abbasi et al. [28], Effat et al. PLR [33], Effat et al. IVF [33], McGregor et al. [34], Helmy et al. [38]	98/173	0.70 (0.56, 0.80)	0.80 (0.50, 0.94)	2.00 (1.56, 2.56)	0.45 (0.34, 0.60)
∆CAVTI	Chowhan et al. [26], Marik et al. [9], Girotto et al. [35], Pace et al. [37]	65/112	0.89 (0.80, 0.94)	0.71 (0.39, 0.90)	5.14 (2.78, 10.61)	0.30 (0.23, 0.42)
CFT	Barjaktarevic et al. [25], Jelic et al. [32], Jalil et al. [39], Abassi et al. [28]	78/153	0.65 (0.52, 0.76)	0.92 (0.61, 0.99)	1.90 (1.48, 2.41)	0.42 (0.29, 0.60)

∆CDPV = change in carotid Doppler peak velocity, CBF = carotid blood flow, ∆CAVTI = change in carotid artery velocity time integral, CFT = carotid flow time, CI = confidence interval.

**Table 5:** Fluid responsiveness for included studies.

Study	Number of patients				Cutoff value	Sensitivity (%)	Specificity (%)	AUROC (95% CI)
	TP	FP	FN	TN				
Barjaktarevic et al. [25]	37	1	17	22	7 mSec	69	96	0.88 (0.80, 0.96)
Chowhan et al. [26]	5	4	2	9	15.8%	71	69	0.69
Zhang et al. [27]	21	6	9	24	11.2 cm/sec	70	80	0.80 (0.69, 0.91)
Abbasi et al. [29]	36	18	14	18	8%	72	50	0.61 (0.48, 0.73)
Abbassi et al. [28] (CBF)	34	26	22	30	>19 ml/min	61	54	0.58 (0.47–0.68)
Abbasi et al. [28] (CFT)	30	25	26	31	6 mSec	54	55	0.59 (0.46–0.65)
Lu et al. [30]	21	2	6	20	13%	78	91	0.91 (0.817, 1.0)
Marik et al. [9]	16	2	1	15	20%	94	88	Not provided
Soliman et al. [31]	11	1	10	8	20%	52	89	0.73 (0.53, 0.93)
Jelic et al. [32]	5	0	1	11	10%	83	100	Not provided
Effat et al. [33] (post PLR)	16	6	4	18	23%	80	75	0.99 (0.98, 1)
Effat et al. [33] (post FC)	14	1	6	23	23%	70	96	0.99 (0.99, 1)
McGregor et al. [34]	9	7	11	6	10%	45	46	Not provided
Girotto et al. [35]	13	4	9	13	8%	90	24	0.68
Ibarra-Estrada et al. [36]	12	5	9	12	14%	87	86	0.88 (0.77, 0.95)
Pace et al. [37] (∆CDPV)	26	4	4	25	>12	82	95	0.91 (0.79, 0.97)
Pace et al. [37] (∆CVTI)	23	1	5	21	>10	89	86	0.92 (0.80, 0.98)
Helmy et al. [38]	25	3	3	19	>17.3	83	100	0.883 (0.78, 0.99)
Jalil et al. [39]	25	0	5	10	>24.6	60	92	0.75 (0.54, 0.96)
D'Arrigo et al. [40]	10	1	8	24	>10.5	53	96.2	0.74 (0.58, 0.91)

∆CDPV = change in carotid Doppler peak velocity, CBF = carotid blood flow, ∆CAVTI = change in carotid artery velocity time integral, CFT = carotid flow time, CI = confidence interval, PLR = passive leg raise, FC = fluid challenge, TP = true positive, FP = false positive, FN = false negative, TN = true negative, AUROC = area under receiver operator curve.

**Table 6:** Subgroup metaregression for pooled carotid US measures.

Parameter	Category	Studies	Sensitivity (C.I.)	*p*. value	Specificity	*p*. value
Index test threshold	10%	10	0.74 (0.65, 0.83)	0.06	0.89 (0.81, 0.97)	0.92
	15%	11	0.71 (0.63–0.80)		0.74 (0.61–0.88)	

Reference measurement	Gold standard	15	0.74 (0.68, 0.81)	0.01	0.85 (0.76, 0.95)	0.12
	Not gold standard	6	0.68 (0.57, 0.79)		0.76 (0.57, 95)	

Type of fluid challenge	IV fluid	16	0.72 (0.65, 0.79)	0.10	0.78 (0.68, 0.89)	0.02
	PLR	5	0.74 (0.60, 0.87)		0.94 (0.87, 1.0)	

Sepsis/septic shock	Yes	7	0.72 (0.61, 0.83)	0.05	0.89 (0.79, 0.99)	0.94
	No	14	0.73 (0.65)		0.79 (0.67, 0.90)	

Gold standard = left ventricular outflow tract velocity time integral or pulmonary artery catheters, PLR = passive leg raise, CI = confidence interval.

## Data Availability

The data used to support the findings of this study are available from the corresponding author upon reasonable request.
